# The challenging management of an assident parotid tumor: a case of solitary plexiform neurofibroma of the parotid facial nerve

**DOI:** 10.1016/j.bjorl.2020.12.007

**Published:** 2021-01-21

**Authors:** Mounir Hmidi, Kaoutar Cherrabi, Mohamed Sinaa, Karim Nadour

**Affiliations:** a Moulay Ismail Military Hospital, Department of Head and Neck Surgery, Meknes, Morocco; b Hassan II University Hospital, Department of Head and Neck Surgery, Fez, Morocco; c Moulay Ismail Military Hospital, Department of Anatomopathology, Meknes, Morocco

## Introduction

Solitary plexiform neurofibroma is a very rare form of tumor, especially for the parotid portion of facial nerve. The differential diagnosis includes mainly neurofibromas and other parotid tumors. The diversity of its clinical and radiological presentations is the main reason for diagnostic and therapeutic challenges.^1^, ^2^, ^3^

## Case report

A 56 year old male presented with a progressively enlarging left parotid mass for 2 years. No pain had been noted, nor any facial asymmetry or weakness. There was no history of anterior trauma or multiple endocrine Neurofibromatosis type 1 (NF1) in the personal or family medical history. Further, no relevant past interventions were noted.

The mass was 2 cm in breadth and 2 cm in width, and adherent to deep and superficial structures, firm, adjacent to the mandibular angle inferiorly and to the ear lobule superiorly. MRI of the parotid region showed a tumor similar to cystic lymphadenoma of the parotid gland. The patient was aware of the risk of facial nerve impairment, and surgery was planned.

**Figure 1:**
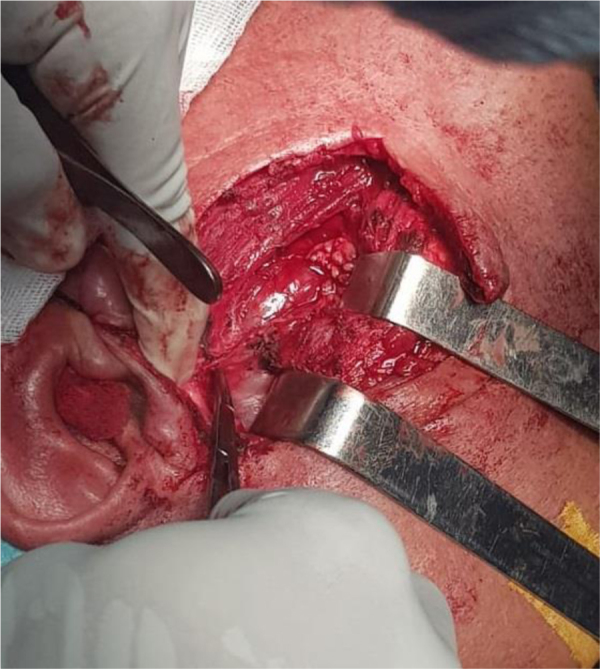
Per-operative aspect of the tumors. Upon dissection of the left parotid gland, the tumor is identified at its origin, at the base of the styloid process.

**Figure 2:**
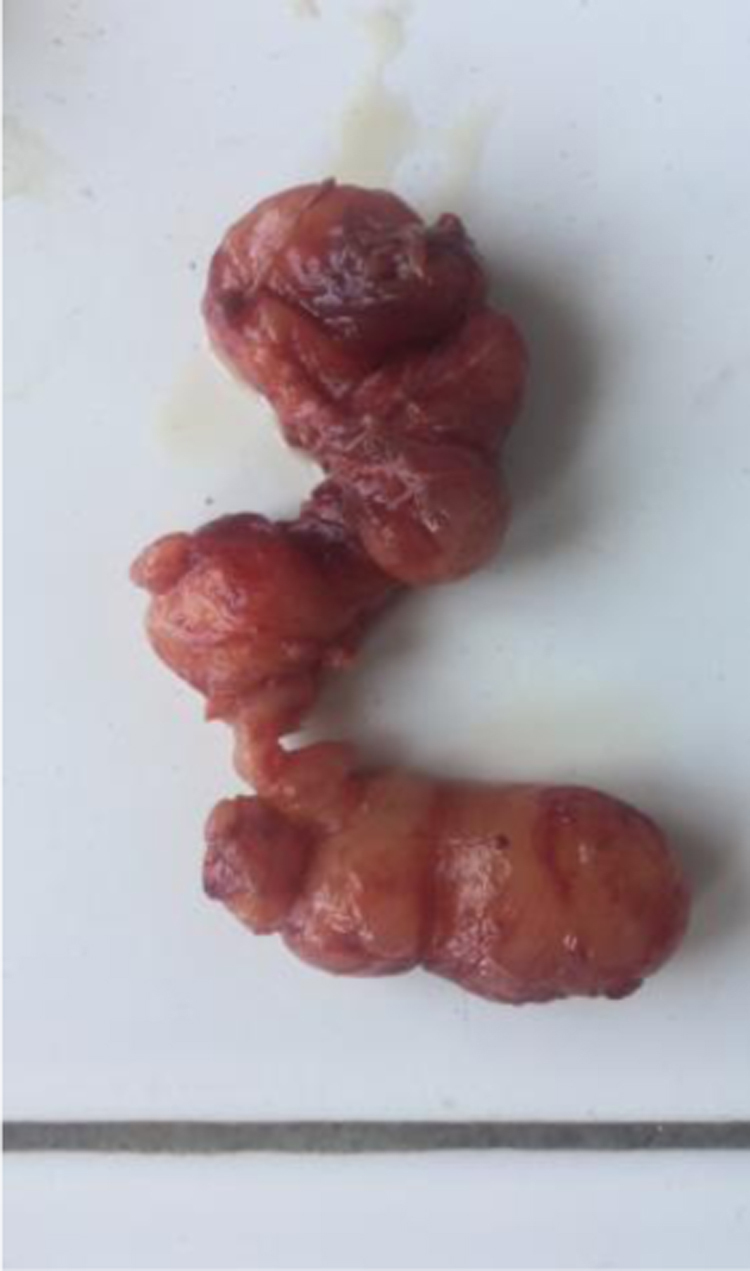
Macroscopic aspect of the parotid tumor. The excision of the tumor was complete.

Peroperative exploration found healthy tissue in cutaneous, subcutaneous, and parotid tissue, and the tumor originated from the emergence point of the trunk of the facial nerve, at the base of the styloid process, (Fig. 1) and extended into the intraparotid portion, up to the anterior edge of the masseter muscle. The complete excision of the tumor involved the excision of facial nerve, with few intact fibers (Fig. 2). Surgical nerve repair was not possible. The immediate postoperative outcome showed stage four House and Brackman peripheral facial nerve palsy.

**Figure 3:**
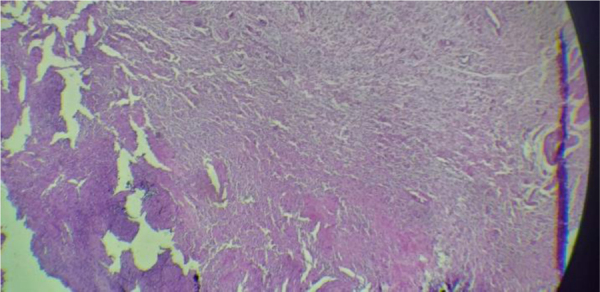
Microscopic sections.

The histological aspect showed the tumor is unencapsulated: It was comprised of a mixture of Schwann cells, peri-neural cells and endoneural fibroblasts (Fig. 3). The diagnosis of plexiform neurofibromatosis was established. The next step consisted of the elimination of other syndromic and nonsyndromic forms. The physical exam showed the absence of “café-au-lait” lesions, of inguinal and femoral freckling. Further all the criteria of neurofibromatosis type I were eliminated, and no other cutaneous or subcutaneous tumor was found.

A body computed tomography (CT) scan was performed and showed no adrenal gland or other abdominal or pelvic tumors. Computed tomodensitometry scan and an magnetic resonance imaging (MRI) of the temporal bone were performed, and showed no trace of schwannomas, or other tumors of cranial nerves.

Prognostic evaluation was based on the surgical outcome and the risk of recurrence and malignant transformation. No change in therapeutic intervention was made after diagnostic confirmation. Assessment of the peripheral facial nerve palsy was established postoperatively, via electromyography.

The patient showed great tolerance towards his facial nerve impairment and faithfully followed ophthalmological protective measures. No adverse or unanticipated events were noted.

## Discussion

Neurogenic tumors of the seventh cranial nerve are rare, and this parotid localization seems to be even rarer. The estimated incidence ranges from 0.2% to 1.5%.^1^, ^2^, ^3^ Solitary plexiform neurofibroma is one of the rarest forms: only three cases of neurofibroma have been cited in the English literature.^1^

It is a tumor depending on a nerve sheet.^4^ Preoperative diagnosis is difficult due to variation in clinical presentation and its close relationship to the nerve site involved.^3^

There is no pathognomonic symptom associated with intraparotid plexiform neurofibroma. Pain with facial motor problems such as paralysis or spasms are mostly associated with this tumor. Positive cytology is scarce, since the cells are well-adherent.^1^, ^4^ No imaging findings are pathognomonic of plexiform neurofibromas.^3^ Neuromas can have a syndromic presentation, as a part of Recklinghausen’s disease, or as multiple neurofibromas without Recklinghausen’s disease, or as solitary neurofibromas.^3^, ^4^

Beside café-au-lait lesions, other cutaneous and subcutaneous tumors or focal neurological signs are also present in most cases.^5^

The diagnosis is established upon personal and familial medical history, as well as clinical and radiological findings. Syndromic neurofibromas are first eliminated, followed by non-syndromic forms.^1^, ^2^, ^6^ The main treatment of solitary plexiform neurofibromas consists of surgical excision.^1^, ^2^, ^3^, ^4^, ^5^, ^6^

In a macroscopic scale, neurofibromas are nonencapsulated and multiple, and generally do not include axons. However, schwannomas are encapsulated, mostly solitary, attached to nerves or surrounded by them. Whereas the neurons present with signs of degeneration, such as necrosis and cystic alterations.^1^, ^4^

However, histologically, neurinomas and schwannomas present some particular characteristics: neurofibromas have much looser structures than schwannomas (Sullivan et al.). In addition, schwannomas are presented in two patterns Antoni A and Antoni B.^3^, ^5^

In Antoni A are found pindle cells, with elongated nuclei arranged in waves, drift, and whorls, which join in braided ribbons. On a cross section, the cylindrical cells are arranged in a palisade-like pattern around the cytoplasm in the center called Verocay body.^3^, ^5^

In Antoni B are very loose tissues, without particular arrangement.^3^, ^5^

In the other hand, neurofibromas are characterized by hardly any organized structure of spindle cells in a loose tissue of collagenous matrix, which contains nerve fibers within the tumor.^3^, ^5^ Followup surveillance is recommended to be yearly as long as the tumor is stable.^5^ When the examination of the lesion shows abnormalities, an MRI and/or positron emission tomography (PET) scan is indicated. If signs of malignancy are found, a biopsy is indicated, and the therapeutic decision is based upon the histological findings.^5^

Although the issue of facial nerve function is central to the treatment of benign neurogenic facial nerve neoplasms, rare malignant tumors need surgical removal with adequate surgical margins.

The presence of a preoperative weakness of facial nerve is an important factor in postoperative prognosis.^1^, ^3^ However, when facial nerve function is intact, controversies about management arise. Some authors recommend avoiding surgery when all parameters indicate the benign nature of the tumor, in which case followup protocols for benign neurogenic tumors seem to be a debatable option, through electro-neurography and radiological imaging.^3^, ^5^

Whenever the nerve function is impaired, malignancy is suspected and further investigation indicated.^3^, ^5^ The risk of malignant transformation in solitary plexiform neurofibroma is less than that of syndromic forms.^1^, ^3^, ^4^ Most of sarcomatous transformations occur in deeply- seated NF1 associated tumors.^1^, ^6^

## Conclusion

Solitary plexiform neuroma is a very rare form of tumor of the parotid portion of facial nerve. Considering the possible functional repercussions of surgical management, surgeons should be aware of its presence in order to prepare a comprehensive plan for management and followup.

## Conflicts of interest

The authors declare no conflicts of interest.
